# Association of the serum uric acid-to-HDL-cholesterol ratio with kidney stones in U.S. adults: A cross-sectional study (NHANES 2007–2016)

**DOI:** 10.1097/MD.0000000000045376

**Published:** 2025-10-24

**Authors:** Jianpeng Yu, Jia Lu, Qianqian Xu, Haiying Zhang, Xinyan Li, Binhua Nian, Rui Liu

**Affiliations:** a Department of Cardiology and Nephrology, The 944 Hospital of the Joint Logistic Support Force, Jiuquan, Gansu, China; b Department of Rheumatology and Immunology, The First Affiliated Hospital of Naval Medical University, Shanghai, China.

**Keywords:** cross-sectional study, kidney stone, NHANES, uric acid to high-density lipoprotein cholesterol ratio

## Abstract

Whether the serum uric acid-to-high-density lipoprotein cholesterol ratio (UHR) is associated with kidney-stone risk remains unknown. This study was a cross-sectional analysis of 11,073 adults aged ≥ 20 years from the National Health and Nutrition Examination Survey (NHANES) 2007 to 2016. Information on kidney stone history was obtained via a self-reported questionnaire. The UHR was calculated as follows: (uric acid mg/dL ÷ high-density lipoprotein cholesterol mg/dL) × 100%. Logistic regression was used to estimate the odds ratio (OR) and 95% confidence interval (95% CI) per one-unit UHR increase and across UHR tertiles, adjusting for sociodemographic factors, comorbidities, and metabolic covariates. Restricted cubic splines were used to determine the dose–response and potential breakpoints; subgroup analyses were conducted to test for interaction effects. The overall kidney stone prevalence was 10.6%. The median UHR was greater in patients with a history of kidney stones than in those without kidney stones (*P* < .001). After multivariable adjustment, each one-unit increase in the UHR was associated with a 2% greater risk of developing kidney stones (OR 1.02; 95% CI: 1.01–1.04). Compared with the lowest tertile, the middle and highest tertiles had 32% (OR 1.32; 95% CI: 1.02–1.71) and 41% (OR 1.41; 95% CI: 1.07–1.87) greater risks of developing kidney stones, respectively. A spline curve suggested a nonlinear relationship with an estimated breakpoint at a UHR of 12.27, but the likelihood-ratio test did not confirm a significant threshold (*P* = .142). Associations were consistent across sex, age, race, and metabolic subgroups (*P*-interaction > .05). In this cross-sectional analysis, a greater UHR was associated with a modestly greater prevalence of self-reported kidney stones.

## 1. Introduction

Kidney stones are a clinical syndrome characterized by the formation of solid crystalline masses in the urinary cavities of the kidneys.^[1]^ The incidence and prevalence of kidney stones, a common and frequently occurring urinary system disorder, are increasing globally, affecting approximately 10 to 15% of the population.^[2,3]^ Without effective prevention and control, the annual kidney stone recurrence rate is expected to reach 10 to 23%, with a 5- to 10-year recurrence rate of up to 50% and a 20-year recurrence rate that can even reach 75%.^[4]^ The long-term presence of kidney stones can lead to clinical manifestations such as urinary tract obstruction, urinary system infections, and a gradual decline in renal function, potentially increasing the risk of end-stage kidney disease and dialysis.^[5]^ These complications significantly impact patients’ daily activities and quality of life. The pathogenesis of kidney stones is not fully understood, but metabolic factors, inflammation, and oxidative stress are believed to play important roles in their development.^[6]^ In recent years, the relationship between metabolic markers and kidney stone incidence has become a research hotspot. For example, the triglyceride-glucose (TyG) index may exacerbate the incidence and recurrence of kidney stones,^[7]^ whereas dietary niacin intake is negatively correlated with kidney stone risk.^[8]^ The uric acid-to-high-density lipoprotein cholesterol ratio (UHR) is an emerging metabolic risk marker that reflects the inflammatory state of the body and oxidative stress levels. In a large-scale study, Kolahi Ahari et al demonstrated that the UHR serves as a novel indicator of inflammation: An increase in the UHR was closely associated with both the presence and severity of metabolic syndrome.^[9]^ Additionally, Yin et al, utilizing National Health and Nutrition Examination Survey (NHANES) 2005 to 2018 data, revealed that the UHR is a significant predictive biomarker for diabetes risk, further underscoring its utility in capturing early metabolic dysfunction.^[10]^ By integrating pro-oxidant uric acid (UA) with anti-inflammatory high-density lipoprotein cholesterol (HDL-C), the UHR offers a more nuanced and stable index of systemic redox imbalance and chronic low-grade inflammation than either component alone. This composite metric positions the UHR as a potentially sensitive tool for identifying the subclinical inflammatory and oxidative milieu implicated in kidney stone formation. Compared with UA or HDL-C levels alone, the UHR provides a more comprehensive reflection of the metabolic status of UA and HDL-C, with greater sensitivity and specificity in predicting diseases such as metabolic syndrome.^[11]^ Previous studies have shown that elevated UA levels increase kidney stone risk, whereas higher HDL-C levels reduce this risk.^[12,13]^ Although the TyG index has been widely used as a surrogate marker of insulin resistance and has been linked to various metabolic disorders, its clinical utility in assessing disease risk – particularly in the context of kidney stones – remains limited. One major drawback is that the TyG index is typically calculated using a single measurement of fasting triglycerides and glucose, which may not accurately reflect long-term metabolic status. Insulin resistance is known to fluctuate over time, and single-point measurements are susceptible to short-term variations caused by acute illness, stress-induced hyperglycemia, or recent dietary intake. In contrast, the UHR, which integrates 2 metabolically stable and pathophysiologically relevant biomarkers, may offer a more consistent and comprehensive reflection of chronic metabolic stress and inflammatory status. As a complex metabolic indicator, the UHR may be used to assess kidney stone risk more accurately. On the basis of this hypothesis, we conducted a cross-sectional study using NHANES data to evaluate the association between the UHR and kidney stone risk, aiming to provide valuable clinical insights for kidney stone prevention.

## 2. Materials and methods

### 2.1. Database selection and the inclusion/exclusion criteria for the study population

**Figure 1.**
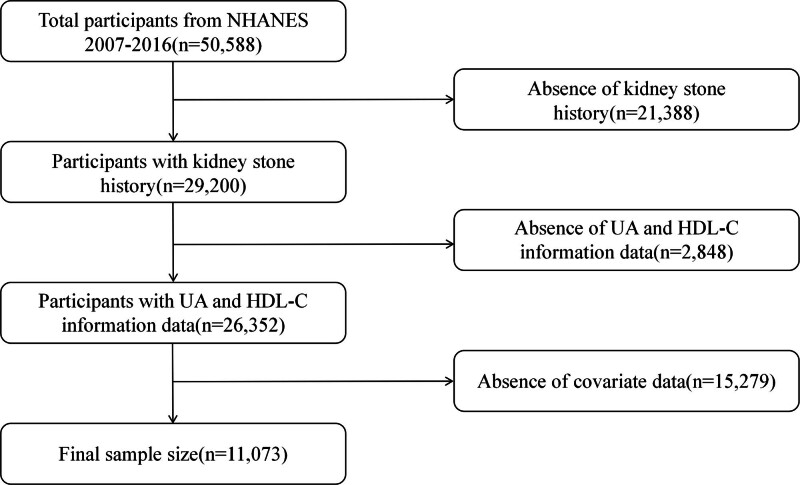
Flow chart of study population inclusion and screening.

The NHANES is a comprehensive and nationally representative survey conducted by the US Centers for Disease Control and Prevention to assess the health and nutritional status of the U.S. population through interviews, physical examinations, and laboratory tests.^[14]^ A total of 50,588 participants were included in the NHANES from 2007 to 2016. Among these participants, 21,388 were excluded because of a lack of data on kidney stone history, 2848 were excluded because of missing clinical measurements of UA and HDL-C, and 15,279 were excluded because of incomplete covariate data. Ultimately, 11,073 participants who met all the criteria were included in the study (Fig. 1). The Ethics Review Board of the National Center for Health Statistics approved all NHANES protocols, the NHANES protocols for the years 2007 to 2008 and 2009 to 2010 were approved under Protocol #2005-06, while the protocols for the years 2011 to 2012, 2013 to 2014, and 2015 to 2016 were approved under Protocol #2011-17. The study utilized data from NHANES, a publicly available dataset. All methodologies were conducted in compliance with pertinent guidelines and regulations, including the Declaration of Helsinki. Prior to study participation, all individuals provided written consent. The datasets analyzed in the present study can be accessed from the NHANES database at https://wwwn.cdc.gov/nchs/nhanes/.

## 3. Measurement of the UHR

UA levels were measured using the time-endpoint method with Beckman UniCel® DxC 800 and Beckman UniCel® DxC 660i automatic biochemical analyzers. HDL-C levels were measured using the Cobas 6000 automatic biochemical immunoassay system. Fasting serum samples were collected, stored at −30°C, and transported to the University of Minnesota for analysis. The detailed laboratory testing methods can be found on the NHANES website (https://wwwn.cdc.gov/nchs/nhanes/analyticguidelines.aspx). The UHR was calculated as the UA level (mg/dL) divided by the HDL-C level (mg/dL) multiplied by 100%.

## 4. Diagnosis of kidney stones

Kidney stone diagnosis was based on a self-reported standardized questionnaire in which the participants were asked, “Have you ever had a kidney stone?” Those who answered affirmatively were considered to have kidney stones, whereas those who answered negatively were considered not to have kidney stones.

## 5. Covariates

Covariates included demographic characteristics, disease history, lifestyle factors, and related clinical laboratory indicators, such as sex, age, race, education level, marital status, hypertension history, diabetes history, coronary heart disease history, smoking history, alcohol consumption history, serum albumin (Alb) level, serum creatinine (Scr) level, fasting blood glucose level, total cholesterol level, triglyceride (TG) level, and low-density lipoprotein cholesterol level. Race was categorized as Mexican American, other Hispanic, non-Hispanic white, non-Hispanic black, or other. Education levels were classified as less than grade 9, grades 9 to 11, high school graduation/GED or equivalent, college or AA degree, and an associate’s degree or above. Marital status was divided into married/living with a partner, widowed/divorced/separated, and never married. The determination of hypertension, diabetes, coronary heart disease, smoking, and alcohol consumption histories was based on self-reported standardized questionnaires.

## 6. Statistical methods

The NHANES employs a complex multistage sampling method to select the study population. Given that the probability of each individual being sampled is not equal and that the sampling data at each stage are not independent, this study assigned corresponding weights to the data to correct for sampling errors during the analysis process. The study population was divided into groups on the basis of kidney stone status. The measurement data are presented as medians (interquartile ranges), and comparisons between groups were made using weighted Mann–Whitney *U* nonparametric tests. Count data are expressed as frequencies (percentages), and weighted χ^2^ tests were used for comparisons. Weighted univariate and multivariate logistic regression analyses were conducted to evaluate the relationships between the UHR and kidney stone risk, adjusting for sex, age, race, marital status, hypertension history, diabetes history, coronary heart disease history, Alb level, Scr level, fasting glucose level, and TG level. The results are presented as odds ratio (OR) and 95% confidence interval (95% CI). The dose–response relationships between the UHR and kidney stone risk and the inflection point were subsequently evaluated using restricted cubic spline plots and threshold effect analyses. Finally, the study population was stratified into different subgroups, and interaction tests were performed to assess heterogeneity between subgroups. Statistical analyses were conducted using R 4.2.3 and EmpowerStats 4.1 software, with *P* < .05 indicating statistical significance.

## 7. Results

### 7.1. Characteristics of the participants

A total of 11,073 participants were included in this study, comprising 5415 males and 5658 females. Among all the participants, 1061 had a history of kidney stones, whereas 10,012 did not. The overall prevalence of kidney stones was 10.6%. Table 1 provides an overview of the general information of the participants in the kidney stone and kidney stone-free groups. Significant differences were observed between the 2 groups in terms of the UHR, sex, age, race, marital status, hypertension history, diabetes history, coronary heart disease history, and Alb, Scr, fasting blood glucose, TG, UA, and HDL-C levels. The UHR was significantly greater in the kidney stone group than in the kidney stone-free group. No significant differences were found in the total cholesterol level, low-density lipoprotein cholesterol level, education level, smoking history, or alcohol consumption history between the groups.

**Table 1:** Characteristics of NHANES participants, 2007–2016.

Characteristics	Non-kidney stone	Kidney stone	*P*-value
N	10,012	1061	
UHR	10.20 (7.37–13.78)	11.57 (8.50–14.90)	<.0001
Age (yr)	47 (33–60)	54 (42–65)	<.0001
HDL-C (mg/dL)	52 (43–64)	47 (41–59)	<.0001
TC (mg/dL)	190 (164–217)	190 (166–216)	.3837
Alb (g/dL)	4.30 (4.10–4.50)	4.20 (4.00–4.40)	<.0001
Scr (mg/dL)	0.83 (0.71–0.98)	0.88 (0.74–1.02)	.0320
UA (mg/dL)	5.40 (4.50–6.30)	5.60 (4.60–6.60)	.0010
TG (mg/dL)	101 (70–146)	110 (78–166)	.0002
LDL-C (mg/dL)	111 (90–136)	113 (91–136)	.8353
GLU (mg/dL)	99 (92–108)	103 (94–115)	<.0001
Education level (%)			
<9th grade	1052 (5.58%)	102 (4.76%)	.1509
9–11th grade	1468 (11.25%)	170 (11.99%)	
High school graduate	2246 (21.88%)	241 (22.28%)	
Some college or AA degree	2815 (30.57%)	331 (33.95%)	
College graduate or above	2431 (30.73%)	217 (27.02%)	
Marital status (%)			
Married/Living with partner	5928 (63.54%)	703 (71.35%)	<.0001
Widowed/Divorced/Separated	2177 (17.81%)	272 (20.86%)	
Never married	1907 (18.65%)	86 (7.79%)	
Drinking history (%)			
Yes	7186 (77.00%)	752 (74.86%)	.2396
No	2826 (23.00%)	309 (25.14%)	
Hypertension (%)			
Yes	3553 (32.06%)	536 (47.89%)	<.0001
No	6459 (67.94%)	525 (52.11%)	
Diabetes (%)			
Yes	1170 (8.77%)	223 (16.44%)	<.0001
No	8842 (91.23%)	838 (83.56%)	
Coronary heart disease (%)			
Yes	380 (3.28%)	90 (7.36%)	<.0001
No	9632 (96.72%)	971 (92.64%)	
Sex (%)			
Male	4828 (48.06%)	587 (53.99%)	.0015
Female	5184 (51.94%)	474 (46.01%)	
Race (%)			
Mexican American	1540 (8.44%)	143 (6.21%)	<.0001
Other Hispanic	1119 (5.80%)	132 (5.45%)	
Non-Hispanic White	4282 (68.03%)	588 (78.06%)	
Non-Hispanic Black	2038 (10.72%)	122 (4.79%)	
Other Race	1033 (7.01%)	76 (5.50%)	
Smoking history (%)			
Yes	4463 (44.83%)	515 (48.45%)	.1615
No	5549 (55.17%)	546 (51.55%)	

Value in continuous variables are median (Q1–Q3) and frequency (percentage) for categorical variables.

Alb = serum albumin, GLU = fasting blood-glucose, HDL-C = high-density lipoprotein cholesterol, LDL-C = low-density lipoprotein cholesterol, NHANES = National Health and Nutrition Examination Survey, Scr = serum creatinine, TC = total cholesterol, TG = triglyceride, UA = blood uric acid, UHR = uric acid to high-density lipoprotein cholesterol ratio.

## 8. Associations between the UHR and kidney stone risk

Univariate and multivariate weighted logistic regression analyses demonstrated a significant positive correlation between the UHR and kidney stones. After adjusting for all confounding factors, each one-unit increase in the UHR was associated with a 2% increase in kidney stone risk (OR: 1.02, 95% CI: 1.01–1.04). Sensitivity analysis was conducted when the UHR was transformed from a continuous variable to a categorical variable (tertile), and the positive correlation persisted across UHR tertile groups (*P* for trend < .05). Compared with that in the lowest tertile (tertile 1), the risk of kidney stones increased by 32% (OR: 1.32, 95% CI: 1.02–1.71) in the middle tertile (tertile 2) and by 41% (OR: 1.41, 95% CI: 1.07–1.87) in the highest tertile (tertile 3). The results are shown in Table 2.

**Table 2:** Associations between the UHR and kidney stone risk.

	Crude model OR (95% CI)	*P*-value	Model 1 OR (95% CI)	*P*-value	Model 2 OR (95% CI)	*P*-value
UHR (continuous)	1.05 (1.03–1.06)	<.0001	1.04 (1.02–1.06)	<.0001	1.02 (1.01–1.04)	.0140
UHR (tertile)						
Tertile 1	Reference	/	Reference	/	Reference	/
Tertile 2	1.49 (1.17–1.89)	.0017	1.48 (1.15–1.90)	.0031	1.32 (1.02–1.71)	.0389
Tertile 3	1.78 (1.42–2.23)	<.0001	1.74 (1.36–2.23)	<.0001	1.41 (1.07–1.87)	.0178
*P* for trend		<.0001		<.0001		.0192

Crude model: Univariate weighted logistic regression analysis. Model 1: Corrected for age, sex, race, marital status; Model 2: Corrected for age, sex, race, marital status, hypertension, diabetes, coronary heart disease, Alb, Scr, TG, GLU.

Alb = serum albumin, CI = confidence interval, GLU = fasting blood-glucose, OR = odds ratio, Scr = serum creatinine, TG = triglyceride, UHR = uric acid-to-high-density lipoprotein cholesterol ratio.

## 9. Restricted cubic spline and threshold effect analysis of the association between the UHR and kidney stone risk

**Figure 2.**
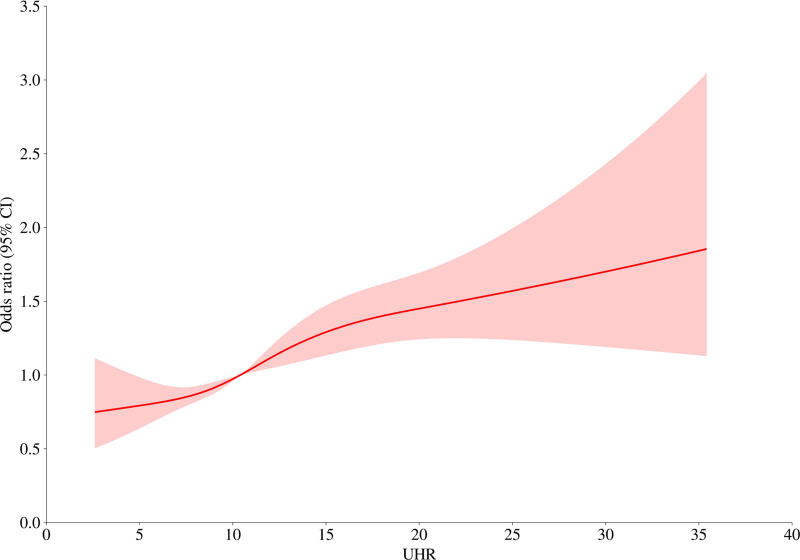
The restricted cubic plots between the UHR and kidney stone risk. The horizontal axis represents the UHR, the vertical axis represents the logical regression OR of the model, the solid red line represents the restricted cubic spline curve, and the shaded part represents the 95% CI. The spline curve was fitted using 4 knots placed at the 5th, 35th, 65th, and 95th UHR percentiles. No threshold effect was found for the association between the UHR and kidney stone risk. Adjusted for age, sex, race, marital status, hypertension history, diabetes history, coronary heart disease history, and Alb, Scr, TG, and GLU levels. Alb = albumin, CI = confidence interval, GLU = glucose, OR = odds ratio, Scr = serum creatinine, TG = triglyceride, UHR = uric acid-to-high-density lipoprotein cholesterol ratio.

Restricted cubic spline analysis and threshold effect analysis were performed to further elucidate the association between the UHR and kidney stone risk, (Fig. 2 and Table 3). The results indicated a nonlinear relationship between the UHR and kidney stone risk, with a breakpoint at 12.27. However, the logarithmic likelihood ratio test (*P* = .142) suggested that the overall association did not exhibit a significant threshold effect.

**Table 3:** Threshold effect analysis of the relationship between the UHR and kidney stone risk.

	Adjusted β (95% CI)	*P* value
Fitting by standard linear model	1.04 (1.03–1.05)	<.001
Fitting by two-piecewise linear model		
Breakpoint (K)	12.27	
β1 (<K)	1.07 (1.03–1.11)	<.001
β2 (>K)	1.02 (1.00–1.05)	.046
Logarithmic likelihood ratio test *P* value		.142

Corrected for age, sex, race, marital status, hypertension, diabetes, coronary heart disease, Alb, Scr, TG, GLU.

Alb = serum albumin, CI = confidence interval, GLU = fasting blood-glucose, Scr = serum creatinine, TG = triglyceride, UHR = uric acid-to-high-density lipoprotein cholesterol ratio.

## 10. Subgroup analysis and interaction tests

Different subgroups were established on the basis of sex, age, race, education level, marital status, hypertension history, diabetes history, coronary heart disease history, smoking history, and alcohol consumption history to further investigate whether the association between the UHR and kidney stone risk was influenced by other factors. The degree of difference in the results of the multivariate weighted logistic regression analysis of Model 2 across different subgroups was observed through interaction tests (Table 4). Importantly, all *P* values for interaction were >.05, indicating consistent null interaction effects across subgroups. This finding reinforces the robustness of our main result, suggesting that the positive association between the UHR and kidney stone risk is generalizable and not significantly modified by demographic or clinical characteristics.

**Table 4:** Subgroup analysis of the association between the UHR and kidney stone risk.

Variable	OR (95% CI)	*P*-value	*P*-interaction
Sex			
Male	1.01 (0.99–1.03)	.2366	.8026
Female	1.01 (0.99–1.04)	.2515	
Age			
˂60	1.01 (0.99–1.03)	.5189	.6064
≥60	1.01 (0.99–1.03)	.1895	
Race			
Mexican American	1.00 (0.97–1.04)	.8178	.0893
Other Hispanic	0.97 (0.93–1.01)	.1508	
Non-Hispanic White	1.02 (1.01–1.04)	.0102	
Non-Hispanic Black	1.00 (0.96–1.03)	.7967	
Other Race	1.02 (0.97–1.07)	.4314	
Education level			
<9th grade	0.98 (0.94–1.02)	.3724	.3373
9–11th grade	1.01 (0.97–1.04)	.7475	
High school graduate	1.00 (0.98–1.03)	.7524	
Some college or AA degree	1.02 (1.00–1.05)	.0491	
College graduate or above	1.02 (0.99–1.05)	.1305	
Marital status			
Married/Living with partner	1.01 (1.00–1.03)	.1209	.7392
Widowed/Divorced/Separated	1.00 (0.98–1.03)	.7724	
Never married	1.02 (0.98–1.07)	.3605	
Smoke			
Yes	1.01 (0.99–1.02)	.5956	.2382
No	1.02 (1.00–1.04)	.0531	
Diabetes			
Yes	1.00 (0.97–1.03)	.9366	.2652
No	1.02 (1.00–1.03)	.0622	
Coronary heart disease			
Yes	1.00 (0.96–1.04)	.8874	.4293
No	1.01 (1.00–1.03)	.0907	
Hypertension			
Yes	1.01 (0.99–1.03)	.2948	.7106
No	1.01 (0.99–1.04)	.1722	
Drink history			
Yes	1.01 (0.99–1.03)	.2607	.6384
No	1.02 (0.99–1.04)	.1969	

Corrected for age, sex, race, marital status, hypertension, diabetes, coronary heart disease, Alb, Scr, TG, GLU.

Alb = serum albumin, CI = confidence interval, GLU = fasting blood-glucose, OR = odds ratio, Scr = serum creatinine, TG = triglyceride, UHR = uric acid-to-high-density lipoprotein cholesterol ratio.

## 11. Supplementary analysis

To evaluate potential selection bias introduced by the exclusion of 15,279 participants with incomplete covariate data, we compared baseline characteristics between the analytic cohort (n = 11,073) and those excluded for missing covariates (Table S1, Supplemental Digital Content, https://links.lww.com/MD/Q405). Kidney stone history did not differ materially between groups (9.58% vs 9.14 %, *P* = .228). The excluded participants were slightly younger (median age 48 vs 50 years, *P* < .001), whereas sex distribution was comparable (48.4% male excluded vs 48.9% included, *P* = .405). These findings suggest that the main determinants of exclusion were data completeness rather than stone-related factors, supporting the generalizability of our results to the broader NHANES population.

In addition, to further contextualize the clinical significance of our primary findings, we estimated the absolute risk increase in kidney stone prevalence associated with elevated UHRs. Based on the baseline prevalence of 10.6% in our study population, each one-unit increase in the UHR corresponded to an estimated 0.21 percentage point increase in absolute risk. Moreover, an individual at the 25th percentile of UHR (~7.4) moving to the 75th percentile (~13.8) would experience an estimated 1.4 percentage point increase in absolute risk (Table S2, Supplemental Digital Content, https://links.lww.com/MD/Q405). Although modest at the individual level, these estimates suggest potential utility of the UHR in population-level risk stratification, particularly given its derivation from routinely available laboratory parameters.

## 12. Discussion

This study is the first to identify a novel association between the UHR and kidney stone risk. Univariate and multivariate weighted logistic regression analyses revealed a significant positive correlation between the UHR and kidney stone risk. In Model 2, which was fully adjusted for confounding factors, each one-unit increase in the UHR was associated with a 2% increase in kidney stone risk. A UHR in the middle tertile (tertile 2) was associated with a 32% increased risk of kidney stones, whereas a UHR in the highest tertile (tertile 3) was associated with a 41% increased risk. Further restricted cubic spline plots revealed a clear nonlinear positive relationship between the UHR and kidney stone risk, and the overall association did not exhibit a significant threshold effect. Additionally, subgroup analysis and interaction tests did not reveal any subgroup factors that affected the positive association between the UHR and kidney stone risk. Therefore, the linear model is favored, and the breakpoint at 12.27 should not be prioritized in the interpretation of the results. UHR could be used as a biomarker to assess the risk of developing kidney stones has important public health implications.

Although previous studies have not directly addressed the role of the UHR in the development of kidney stones, the relationships among UA levels, HDL-C levels, and kidney stone risk have been widely discussed. UA stones are the second most common type of kidney stones and account for approximately 5 to 15% of all kidney stones.^[15]^ A retrospective study of 555 patients with upper urinary calculi revealed that hyperuricemia was an important risk factor for bilateral upper urinary calculus formation.^[16]^ Moreover, patients with urinary calculi presented significantly higher levels of UA than did those without urinary calculi.^[17]^ Another cross-sectional study demonstrated that HDL-C levels were significantly negatively correlated with kidney stone incidence,^[18]^ and the levels of HDL-C in patients with kidney stones were significantly lower than those in individuals without kidney stones.^[19]^ Consistent with previous epidemiological studies that have separately linked dyslipidemia and hyperuricemia to nephrolithiasis, the present analysis demonstrates that their combined representation in the UHR is positively associated with kidney stone risk. NHANES-based investigations have reported higher odds of stone prevalence and recurrence among participants with elevated non-HDL-to-HDL-cholesterol ratios or fatty liver indices, underscoring the relevance of adverse lipid profiles and hepatic steatosis in stone formation.^[20,21]^ Similarly, a population-based study in Iran observed significantly higher UHR in individuals with metabolic syndrome, reinforcing the ratio’s capacity to capture metabolically driven risk.^[22]^ Collectively, these findings support the notion that the UHR can be used as an effective biomarker for assessing kidney stone risk. These findings support the notion that the UHR can be used as an effective biomarker for assessing kidney stone risk.

The formation of kidney stones is a dynamic biomineralization process. When calcium, phosphorus, oxalic acid, UA, and other stone components reach supersaturation, they precipitate from the urine and nucleate locally. After further growth and aggregation, the crystals interact with renal tubular epithelial cells and eventually attach to renal tubules, leading to the formation of kidney stones.^[23]^ Physical and chemical factors play significant roles in kidney stone formation. The increase in UA levels leads to the supersaturation of UA in urine, promoting the formation of urate crystals. The deposition of excessive urate crystals in renal tubules triggers an inflammatory response and induces kidney stone recurrence.^[24]^ Moreover, the formation of urate crystals is closely related to the acidic environment of the urine.^[23]^ An epidemiological study of 24-hour urine samples from 584 adults revealed a significant positive correlation between urine pH and HDL-C levels, with low HDL-C being a risk factor for a urine pH < 5.5.^[25,26]^ Lipid metabolism disorders are also important mechanisms of kidney stone formation. Previous studies have shown that increased residual cholesterol levels and increased dietary fatty acid intake are associated with an increased risk of kidney stones in adults.^[27,28]^ HDL-C is generally considered “good cholesterol” in the body because of its ability to reverse cholesterol transport, which helps reduce cholesterol levels in peripheral tissues and blood circulation, thereby reducing the risk of cardiovascular disease.^[29]^ Additionally, trans fatty acids can lead to a significant reduction in HDL-C levels.^[30]^ These mechanisms may collectively contribute to increased susceptibility to kidney stone formation.

To our knowledge, this is the first study to explore the association between the UHR and kidney stone risk, revealing a positive correlation between the UHR and kidney stone risk in adults in the U.S. This finding revealed a specific inflection point with important public health implications for preventing kidney stones, which is innovative. The UHR, which is an easily obtained biomarker in clinical practice, combines UA and HDL-C levels in the form of a ratio to reflect the metabolic state and inflammation level of the body, offering more comprehensive predictive value. Moreover, we utilized data from a large, nationally representative database, the NHANES, with a standardized protocol for data collection and rigorous adjustment for multiple confounders, minimizing bias and increasing the accuracy of our results. However, this study has certain limitations. First, the NHANES is a cross-sectional study that captures data at a single point in time, limiting its ability to establish temporal relationships between exposures and outcomes. The association between the UHR and kidney stone risk could be subject to reverse causality, making causality uncertain. Second, the determination of kidney stone history in this study relied on self-reported data, which may introduce recall bias due to the omission of past medical history and affect the validity and reliability of the study results. Third, our study population was limited to adults in the U.S., and the characteristics or behaviors of the sample were not fully representative of all target populations, thus limiting the generalizability of the findings. Additionally, certain demographic and disease history variables not included in this study may have introduced confounding effects. Future longitudinal and mechanistic studies are needed to better control for confounding factors and elucidate the potential mechanisms underlying the effects of the UHR on kidney stone risk. In addition, although we adjusted for an extensive set of sociodemographic, comorbid, and laboratory covariates, BMI, a well-established determinant of both metabolic dysregulation and nephrolithiasis, was not included in the final multivariate models. Consequently, residual confounding by BMI and obesity-related pathways, such as visceral adiposity or insulin resistance, cannot be excluded. Unmeasured variations in dietary sodium, animal protein, and fluid intake, together with the concomitant use of uric acid- or lipid-lowering agents like allopurinol, febuxostat, statins, or fenofibrate, may further distort the reported associations. In the NHANES, however, 24 hours dietary recalls are available only for selected cycles and reflect short-term rather than habitual intake, while medication use is self-reported without dose-duration information. These constraints precluded their inclusion in the primary analysis.

In this cross-sectional study using nationally representative data from NHANES 2007 to 2016, we observed that a greater UHR was statistically associated with a self-reported history of kidney stones among U.S. adults. After adjustment for a range of sociodemographic and metabolic factors, the UHR remained positively associated with kidney stone prevalence. However, due to the cross-sectional design, this analysis cannot establish temporal relationships or infer causality. Further longitudinal and mechanistic studies are warranted to clarify whether the UHR plays any etiologic role in stone formation and to evaluate its potential utility in risk stratification.

## Acknowledgments

The authors express their gratitude to the National Center for Health Statistics for providing access to the NHANES data.

## Author contributions

**Conceptualization:** Jianpeng Yu.

**Data curation:** Qianqian Xu.

**Investigation:** Haiying Zhang.

**Methodology:** Xinyan Li.

**Software:** Binhua Nian.

**Writing – original draft:** Jia Lu.

**Writing – review & editing:** Rui Liu.

## Supplementary Material



## References

[1] Shastri S Patel J Sambandam KK Lederer ED. Kidney stone pathophysiology, evaluation and management: core curriculum 2023. Am J Kidney Dis. 2023;82:617–34. 37565942 10.1053/j.ajkd.2023.03.017 PMC11370273

[2] Dai X Chang Y Hou Y. Associations between the conicity index and kidney stone disease prevalence and mortality in American adults. Sci Rep. 2025;15:902. 39762499 10.1038/s41598-025-85292-9 PMC11704305

[3] Balawender K Łuszczki E Mazur A Wyszyńska J. The multidisciplinary approach in the management of patients with kidney stone disease-a state-of-the-art review. Nutrients. 2024;16:1932. 38931286 10.3390/nu16121932 PMC11206918

[4] Moe OW. Kidney stones: pathophysiology and medical management. Lancet. 2006;367:333–44. 16443041 10.1016/S0140-6736(06)68071-9

[5] Yuan T Xia Y Li B. Gut microbiota in patients with kidney stones: a systematic review and meta-analysis. BMC Microbiol. 2023;23:143. 37208622 10.1186/s12866-023-02891-0 PMC10197343

[6] Wigner P Grębowski R Bijak M Szemraj J Saluk-Bijak J. The molecular aspect of nephrolithiasis development. Cells. 2021;10:1926. 34440695 10.3390/cells10081926 PMC8393760

[7] Qin Z Zhao J Geng J Chang K Liao R Su B. Higher triglyceride-glucose index is associated with increased likelihood of kidney stones. Front Endocrinol. 2021;12:774567. 10.3389/fendo.2021.774567 PMC8667164 34912299

[8] Wu J. Association between dietary niacin intake and kidney stones in American adults. Sci Rep. 2025;15:5666. 39955312 10.1038/s41598-025-87227-w PMC11829973

[9] Kolahi Ahari R Mansoori A Sahranavard T. Serum uric acid to high-density lipoprotein ratio as a novel indicator of inflammation is correlated with the presence and severity of metabolic syndrome: a large-scale study. Endocrinol Diabetes Metab. 2023;6:e446. 37605374 10.1002/edm2.446 PMC10638626

[10] Yin J Zheng C Lin X. The potential of the serum uric acid to high-density lipoprotein cholesterol ratio as a predictive biomarker of diabetes risk: a study based on NHANES 2005–2018. Front Endocrinol. 2024;15:1499417. 10.3389/fendo.2024.1499417 PMC11798810 39916754

[11] Yu X Sun F Ming J. Serum uric acid to high-density lipoprotein cholesterol ratio is a promising marker for identifying metabolic syndrome in nondiabetic Chinese men. Postgrad Med. 2023;135:741–9. 37750609 10.1080/00325481.2023.2263372

[12] Ferraro PM Curhan GC. Serum uric acid and risk of kidney stones. Am J Kidney Dis. 2017;70:158–9. 28739125 10.1053/j.ajkd.2017.05.004

[13] Hung JA Li CH Geng JH Wu DW Chen SC. Dyslipidemia increases the risk of incident kidney stone disease in a large taiwanese population follow-up study. Nutrients. 2022;14:1339. 35405952 10.3390/nu14071339 PMC9000795

[14] Sobus JR DeWoskin RS Tan YM. Uses of NHANES biomarker data for chemical risk assessment: trends, challenges, and opportunities. Environ Health Perspect. 2015;123:919–27. 25859901 10.1289/ehp.1409177 PMC4590763

[15] Huang R Jiang MJ Chen JC. Flexible ureteroscopy combined with potassium sodium hydrogen citrate (PSHC) intervention improves the stone-free rate (SFR) for 20–30 mm uric acid renal stones. BMC Urol. 2025;25:29. 39955501 10.1186/s12894-025-01710-0 PMC11829543

[16] Zheng Z Hu W Ji C. A study of the difference in biochemical metabolism between patients with unilateral and bilateral upper urinary tract stones. Sci Rep. 2024;14:30154. 39627370 10.1038/s41598-024-81454-3 PMC11615316

[17] Li CC Chien TM Wu WJ Huang CN Chou YH. Uric acid stones increase the risk of chronic kidney disease. Urolithiasis. 2018;46:543–7. 29492591 10.1007/s00240-018-1050-1

[18] Liu H Jin M Hao H Yuan Y Jia H Zhou Y. Association between relative fat mass and kidney stones in American adults. Sci Rep. 2024;14:27045. 39511356 10.1038/s41598-024-78061-7 PMC11543931

[19] Du YZ Dong QX Hu HJ. A cross-sectional analysis of the relationship between the non-high density to high density lipoprotein cholesterol ratio (NHHR) and kidney stone risk in American adults. Lipids Health Dis. 2024;23:158. 38802797 10.1186/s12944-024-02150-9 PMC11129406

[20] Hong H He Y Gong Z Feng J Qu Y. The association between non-high-density lipoprotein cholesterol to high-density lipoprotein cholesterol ratio (NHHR) and kidney stones: a cross-sectional study. Lipids Health Dis. 2024;23:102. 38615008 10.1186/s12944-024-02089-x PMC11015599

[21] Zhang F Li W. Association between the fatty liver index, metabolic dysfunction-associated steatotic liver disease, and the risk of kidney stones. Kidney Blood Pressure Res. 2025;50:115–30. 10.1159/000543404 PMC11844708 39746337

[22] Yazdi F Baghaei MH Baniasad A Naghibzadeh-Tahami A Najafipour H Gozashti MH. Investigating the relationship between serum uric acid to high-density lipoprotein ratio and metabolic syndrome. Endocrinol Diabetes Metab. 2022;5:e00311. 34705333 10.1002/edm2.311 PMC8754234

[23] Ferraro PM Bargagli M Trinchieri A Gambaro G. Risk of kidney stones: influence of dietary factors, dietary patterns, and vegetarian-vegan diets. Nutrients. 2020;12:779. 32183500 10.3390/nu12030779 PMC7146511

[24] Asahina Y Sakaguchi Y Oka T. Association between urinary uric acid excretion and kidney outcome in patients with CKD. Sci Rep. 2024;14:5119. 38429496 10.1038/s41598-024-55809-9 PMC10907602

[25] Otsuki M Kitamura T Goya K. Association of urine acidification with visceral obesity and the metabolic syndrome. Endocr J. 2011;58:363–7. 21441701 10.1507/endocrj.k10e-319

[26] Cho YH Lee SY Jeong DW. The association between a low urine pH and the components of metabolic syndrome in the Korean population: findings based on the 2010 Korea National Health and Nutrition Examination Survey. J Res Med Sci. 2014;19:599–604. 25364357 PMC4214016

[27] Wang DW Shi F Zhang DG Wang H Zhu Y Wang J. Remnant cholesterol increases the risk of incident kidney stones: a nested case-control study in Chinese adults. Urolithiasis. 2024;52:160. 39540945 10.1007/s00240-024-01658-0

[28] Tan N Zhang Y. Associations between dietary fatty acids and kidney stones. Sci Rep. 2025;15:2500. 39833367 10.1038/s41598-025-86850-x PMC11747447

[29] Pownall HJ Rosales C Gillard BK Gotto AM Jr. High-density lipoproteins, reverse cholesterol transport and atherogenesis. Nat Rev Cardiol. 2021;18:712–23. 33833449 10.1038/s41569-021-00538-z

[30] Brouwer IA Wanders AJ Katan MB. Effect of animal and industrial trans fatty acids on HDL and LDL cholesterol levels in humans –a quantitative review. PLoS One. 2010;5:e9434. 20209147 10.1371/journal.pone.0009434 PMC2830458

