# Supplementary material for: Association of the serum uric acid-to-HDL-cholesterol ratio with kidney stones in U.S. adults: A cross-sectional study (NHANES 2007–2016)
Source: Medicine (Baltimore). 2025 Oct 24;104(43):e45376. doi: 10.1097/MD.0000000000045376 (PMC12558274; doi:10.1097/MD.0000000000045376)
Supplement: Supplementary file 1 [file medi-104-e45376-s001.docx]

**Association of the serum uric acid-to-HDL-cholesterol ratio with kidney stones in U.S. adults: a cross-sectional study (NHANES 2007–2016)**

Yu, Jianpeng MM^1,#^, Lu, Jia MM^1,#^, Xu, Qianqian BS^2,#^, Zhang, Haiying BS^1^, Li, Xinyan BS^1^, Nian, Binhua BS^1^, Liu, Rui BS^1,*^

^#^Yu, Jianpeng, Lu, Jia, and Xu, Qianqian contributed equally to the work and should be regarded as co-first authors.

**Table S1 Comparison of baseline characteristics between included participants and those excluded due to missing covariate data in the NHANES 2007–2016 analytic sample**

| Characteristics | Missing covariate data | Included participants | P-value |
| --- | --- | --- | --- |
| N | 15279 | 11073 |  |
| kidney stone history (%) |  |  | .2280 |
| No | 13882 (90.86%) | 10012 (90.42%) |  |
| Yes | 1397 (9.14%) | 1061 (9.58%) |  |
| Age (years) | 48 (34 - 63) | 50.00 (35 - 64) | <.0010 |
| Sex (%) |  |  | .4050 |
| Male | 7393 (48.38%) | 5415 (48.90%) |  |
| Female | 7886 (51.62%) | 5658 (51.10%) |  |

Value in continuous variables are median (Q1-Q3) and frequency (percentage) for categorical variables.

**Table S2 Estimated Absolute Risk Increase in Kidney Stone Prevalence by UHR Levels**

| UHR Change Scenario | UHR Value (approx.) | OR (per unit) | Relative Risk Increase | Absolute Risk Increase^*^ |
| --- | --- | --- | --- | --- |
| Per 1-unit increase in UHR | — | 1.02 | 2.0% | +0.21 percentage points |
| From 25th percentile to 75th percentile | ~7.4 → ~13.8 | 1.02 | 13.5% | +1.4 percentage points |

^*^Based on a baseline kidney stone prevalence of 10.6% in the study population.
